# Identification of the Pathogen *Dorcadia ioffi Smit* and Evaluation of the Effect of Different Drugs

**DOI:** 10.3390/vetsci12070641

**Published:** 2025-07-04

**Authors:** Xin Li, Zihang Qin, Haiyan Wang, Jiangtao Xia, Yukang Zhao, Xuelian Ma, Na Li, Gang Yao

**Affiliations:** 1 College of Veterinary Medicine, Xinjiang Agricultural University, Urumqi 830052, China; 320220061@xjau.edu.cn (X.L.); 18699177078@163.com (Z.Q.); maxuelian@xjau.edu.cn (X.M.); nali@edu.xjau.cn (N.L.); 2 Center of Diagnosis and Control for Animal Diseases in Aletai Prefecture, Aletai 836500, China; wanghaiy1977@163.com (H.W.); 18997798685@163.com (J.X.); 18809066861@163.com (Y.Z.)

**Keywords:** Altai sheep, *Dorcadia ioffi Smit*, morphological analysis, fleas, pharmacological efficacy assay

## Abstract

Vermipsyllid parasitize the body surface of sheep, feeding on blood and transmitting diseases, causing severe economic losses to the livestock industry. An outbreak of sheep Vermipsyllid fleas in the mountain pastures of Xinjiang’s Altai region showed that several commonly used commercial anthelmintic drugs had poor therapeutic effects on the prevalent flea species. This study first conducted species identification of Vermipsyllid through morphological analysis of 200 female and 40 male specimens collected from the Altai region, followed by molecular biological identification of 6 randomly selected fleas (3 females and 3 males). Finally, pharmacodynamic experiments were performed to screen for highly effective anthelmintic drugs. Ninety Altai multiparous ewes infected with Vermipsyllid were divided into six groups (fifteen sheep per group): an untreated control group (Ctr), avermectin injection group (Group I), ivermectin injection group (Group II), moxidectin pour-on group (Group III), cypermethrin pour-on group (Group IV), and dichlorvos pour-on group (Group V), with a 14-day trial period. On Day 0, each group received a single treatment according to drug instructions and specified dosages. The number of fleas, flea population reduction rates, and cure rates were measured on Day 0, Day 3, Day 7, and Day 14 to screen for effective anthelmintic drugs. Results showed that unfed female and male fleas were grayish-brown. Engorged females reached 14.15 mm in size, appearing pale white or yellow, with their sterna maintaining the original size and shape despite abdominal distension. Female fleas had 20–21 segments on the labium, while males had 16–19 segments. The hind tibiae featured four notches, and each side of the tergite had 13 ± 1 cup-like indentations. Molecular biological identification indicated that the identified fleas belonged to *D. ioffi* (Vermipsyllidae, *Dorcadia Ioff*), showing 99.13% sequence similarity with *D. ioffi* from Xinjiang, China. In pharmacodynamic experiments, the number of *D. ioffi* in Groups IV and V decreased to zero on both Day 7 and Day 14, which was extremely significantly lower than other groups (*p* < 0.01). The flea population reduction rates in Groups IV and V reached 100% on both days. By Day 14, the cure rates of Groups I, II, and III were 0%, while those of Groups IV and V were 100%. Avermectin injection, ivermectin injection, and moxidectin pour-on showed poor anthelmintic effects, whereas cypermethrin and dichlorvos pour-on exhibited high anthelmintic activity against this flea species.

## Simple Summary

Vermipsyllid parasitize the surface of livestock, feeding on blood and transmitting diseases, which causes severe economic losses to the livestock industry. An outbreak of sheep Vermipsyllid fleas occurred in the mountain pastures of the Altai region, Xinjiang, with clinical treatment revealing significant resistance of this flea species to multiple commonly used anthelmintic drugs. This study identified the local Vermipsyllid species through morphological observation and molecular biological analysis and compared the deworming effects of different drugs via pharmacodynamic experiments. The results showed that the prevalent local species was *Dorcadia ioffi Smit (D. ioffi)*; cypermethrin and dichlorvos exhibited high insecticide activity. This study clarified the species of sheep Vermipsyllid in the region, screened highly effective anthelmintic drugs, and provided a scientific basis for the diagnosis of such parasitic diseases, selection of anthelmintic drugs in pastures, and reduction in drug resistance risks and prevention and control.

## 1. Introduction

The Vermipsyllidae family, belonging to the order Siphonaptera (fleas), class Insecta, and phylum Arthropoda, consists of fully metamorphosizing insects, with a life cycle that includes eggs, larvae, pupae, and adults [1]. After parasitization, adult fleas consume large amounts of blood, with individuals growing rapidly from 4 mm to 16 mm in size. Studies have reported that a single animal can be infected with more than 500 fleas, leading to significant blood loss, which can cause weight loss, anemia, and even death [2,3], resulting in substantial economic losses to the pastoral industry. The Altai region is located at the northernmost tip of Xinjiang, China, bordering Kazakhstan and Russia to the northwest and Mongolia to the northeast, with a border length of 1205 km [4]. Pastoral farming is the main mode of livestock production in the Altai region, which is one of China’s major regions producing sheep meat products for export, with an estimated sheep population of approximately 3 million [5,6]. Parasitic diseases are among the major constraints on the development of pastoral farming in this area [7,8].

In January 2024, an outbreak of Vermipsyllidae infestation in sheep occurred in the winter pastures of the Altai region, Xinjiang. Clinical treatments demonstrated that several commercially available macrocyclic lactones showed poor efficacy against the prevalent flea species. Morphological identification enables rapid preliminary diagnosis of this parasitic disease, while molecular biological identification allows for precise species-level determination and provides a basis for studying drug-resistant genes. Additionally, pharmacological efficacy assays for screening high-efficiency drugs can offer effective treatment regimens, optimize dosing, and mitigate the development of drug resistance. This study employed morphological and molecular biological methods for pathogen identification and conducted pharmacological efficacy assays to screen for potent anthelmintics, aiming to provide scientific evidence for the precise prevention and control of this parasitic disease.

## 2. Materials and Methods

All procedures were approved by the Animal Welfare and Ethics Committee of Xinjiang Agricultural University, with the approval protocol code 2023049 and the approval date 15 April 2023.

### 2.1. Experimental Animals

On 19 January 2024, Vermipsyllidae naturally infecting sheep in the winter pastures of the Altai region, including 200 female and 40 male fleas, were collected for identification. Additionally, 90 Altai ewes (weighing 45 ± 3.8 kg and that had not received deworming treatment in the past six months) naturally infected with Vermipsyllidae were randomly selected for pharmacodynamic experiments.

### 2.2. Main Chemicals

The chemicals used included bifenthrin solution (1 g/100 mL, purchased from Wuhan Wusi Dongbao Pharmaceutical Co., Ltd., Batch number: 23080211,and sourced from Wuhan, China.); carbaryl solution (30%, purchased from Fengcheng Animal Pharmaceutical Co., Ltd., Batch number: 20220819, and sourced from Liaoning, China.); ivermectin injection (0.01 g/mL, purchased from Shanghai Gongyi Pharmaceutical Co., Ltd., Batch number: 230813, and sourced from Shanghai, China.); acetylamino avermectin injection (0.01 g/mL, purchased from Hebei Weiyuan Pharmaceutical Co., Ltd., Batch number: 23012307, and sourced from Hebei, China.); and moxidectin pour-on (0.5 g/100 mL, purchased from Anhui Tianan Biotechnology Co., Ltd., Batch number: 20230120, and sourced from Anhui, China.).

### 2.3. Morphological Identification

In accordance with the methods used to identify fleas described by Li F and Zhao S S [9,10], Vermipsyllidae were collected and fixed in 75% ethanol, with 30 fleas stored per 100 mL of fixative. The fixed specimens were examined as follows:

Direct Observation: Fifteen females and fifteen males were randomly selected for observation, recording, and photographing under an automatic stereomicroscope (M205C, Leica, Wetzlar, Germany). The procedure was as follows: Worms were clamped with forceps, placed on a slide, and positioned on the observation stage. After adjusting the illumination and lens for focusing, images were captured and data were recorded.

Vermipsyllidae were removed from the fixative and washed with distilled water. The specimens were boiled in 10% potassium hydroxide solution for 3–6 min, dehydrated in a gradient alcohol series for 6 min, and clarified in xylene for 2 min. The whole flea or part of the flea was placed on a glass slide, covered with Canada balsam, and observed under a stereomicroscope. Photographs were taken and recorded.

### 2.4. Molecular Biological Identification

On the basis of the molecular identification methods for fleas by Mauchline T H and Yang X [11,12], the following steps were conducted:

The forward primer (LCO1490) and reverse primer (HCO2198) were designed using Primer Premier 5 software, targeting the cytochrome oxidase subunit I (COI) gene partial coding sequence (cds) within the mitochondrial genome of *Dorcadia ioffi*. Specifically, these primers were designed based on the reference sequence of *D. ioffi* isolate W1 (GenBank accession: JQ737066.1; mitochondrial genome, partial cds). The LCO1490 sequence was 5′-GGTCAACAAATCATAAAGATATTGG-3′ and the HCO2198 sequence was 5′-TAAACTTCAGGGTGACCAAAAAATCA-3′. The primers were 18–30 bp in length, with a Tm value between 55 and 65 °C and a GC content of 40–70%, predicted to amplify a ~700 bp fragment of the COI gene across Vermipsyllidae species.

DNA Extraction: Six Vermipsyllidae (three males and three females) were randomly collected. The genomic DNA was extracted following the kit instructions, and the DNA concentration and purity were measured using a spectrophotometer to confirm the quality of the DNA samples.

PCR amplification was performed using a 25 μL reaction system containing 1 μL of template DNA, 1 μL each of 10 μM forward and reverse primers, 1 μL of 10 mM dNTP Mix, 2.5 μL of 10 × Taq Buffer (with MgCl_2_), 0.2 μL of Taq Polymerase (5 U/μL), and ddH_2_O to make up the volume. The amplification conditions comprised pre-denaturation at 95 °C for 5 min, followed by 10 cycles of denaturation at 94 °C for 30 s, annealing at 63 °C with a 0.5 °C decrease per cycle for 30 s, and extension at 72 °C for 30 s, and then 30 cycles of denaturation at 95 °C for 30 s, annealing at 58 °C for 30 s, and extension at 72 °C for 30 s, a final extension at 72 °C for 10 min, and holding at 4 °C.

Gel Electrophoresis: PCR products were analyzed by 1% agarose gel electrophoresis with the electrophoresis parameters set to 150 V and 100 mA for 10–20 min. The band size and concentration were observed.

Gel Purification: PCR product bands were excised from 1% agarose gels and purified using the SanPrep Column-based DNA Gel Recovery Kit (Sangon Biotech., Shanghai, China) according to the manufacturer’s protocols. Sequencing was performed on an ABI 3730XL sequencer. The obtained gene sequences were used to retrieve homologous sequences via NCBI. The data were then imported into MEGA 11 software, where sequence alignment was performed using the Align by ClustalW method. Subsequently, a phylogenetic tree was constructed using the neighbor-joining method with the following parameter settings: the Bootstrap method was set to 1000 replicates to assess branch reliability, the p-distance model was selected under model/method, and a 50% site coverage cutoff was applied to handle gaps in the alignment.

### 2.5. Pharmacodynamics Experiment

Ninety Altai ewes were randomly assigned to six groups of fifteen animals each: an untreated control group (Ctr), avermectin injection group (I), ivermectin injection group (II), moxidectin pour-on group (III), deltamethrin solution pour-on group (IV), and trichlorfon pour-on group (V). Specific treatment regimens and dosages are detailed in Table 1. For the pour-on groups (deltamethrin and trichlorfon), solutions were diluted with warm water and applied topically to ensure full skin contact. Injection groups received a single subcutaneous administration in the neck using a 1 mL syringe (needle gauge 0.45 × 15 mm) according to manufacturer instructions.

On Days 0, 3, 7, and 14, twelve observers were divided into six teams of two. One member of each team restrained the ewe and recorded data, while the other counted Vermipsyllidae within three 10-cm^2^ areas (one each on the neck, chest, and hindquarters) of each animal. The observation time was 10 min per ewe, with each observation session lasting 2.5 h. Ewes were group-grazed throughout the experiment [13,14]. Flea reduction rate and cure rate were calculated using the following formulas [15].

Flea reduction rate (%) = (Initial flea count − Final flea count)/Initial flea count × 100%

Cure rate (%) = (number of ewes with no fleas after treatment/number of ewes with fleas before treatment) × 100%

### 2.6. Data Processing and Statistical Analysis

The number of flea infections in each group was analyzed by one-way ANOVA using GraphPad Prism 10.0.1. Multiple comparisons were performed.

## 3. Results

### 3.1. Parasitic Characteristics and Infection Phenotypes

In this study, Vermipsyllidae were found to be prevalent in mountain pastures at an altitude of 1500–2100 m. They primarily parasitize the skin of sheep’s necks, chests, and buttocks. These parasites consume large amounts of blood. When the wool is parted, yellowish-white parasites the size of soybeans can be observed, surrounded by numerous black granular feces excreted by the parasites and white eggs. Due to the non-coagulation of the wounds at the blood-sucking sites, blood oozes out. In severely infected areas, the wool can be dyed red.

### 3.2. Morphological Result

**Figure 1:**
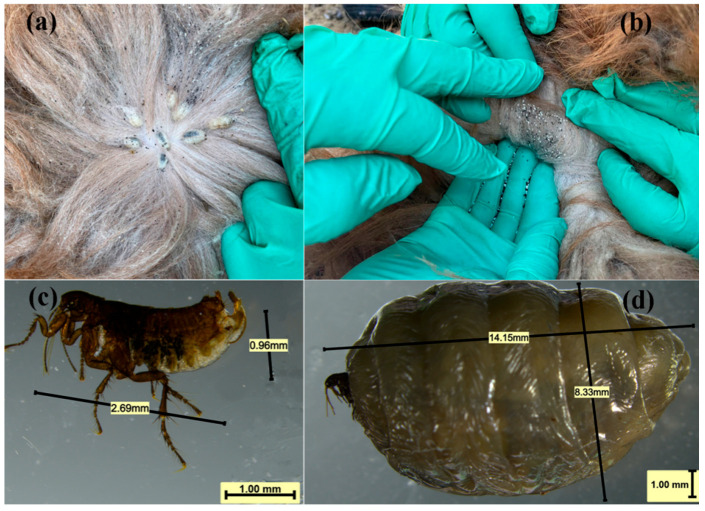
Morphological characteristics of Vermipsyllidae: (**a**) Vermipsyllidae parasite on the sheep body surface; (**b**) eggs laid by female Vermipsyllidae; (**c**) male Vermipsyllidae; and (**d**) female Vermipsyllidae.

**Figure 2:**
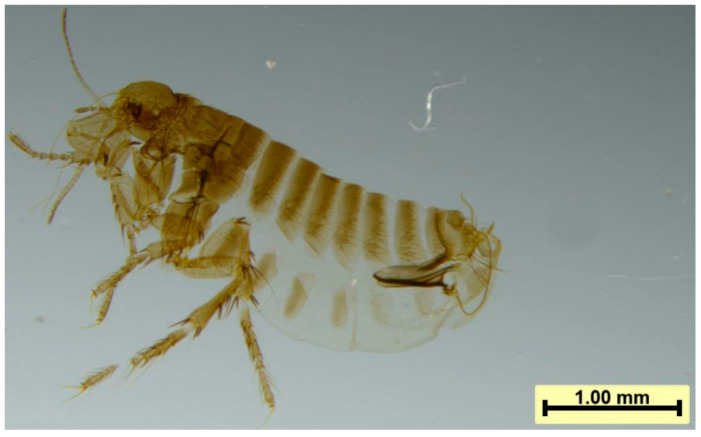
The morphological characteristics of Vermipsyllidae.

In this study, Vermipsyllidae were found to parasitize primarily the neck skin of sheep (Figure 1a). The female fleas expelled large numbers of eggs while feeding on blood (Figure 1b). Microscopic observation revealed that the males were between 2.68 mm and 3.10 mm in length (Figure 1c), whereas the blood-engorged females reached 14.15 mm in length (Figure 1d). The body of the flea was gray-brown in color; however, the body of the engorged female contained numerous eggs, swelled and turned white or pale yellow. The body consisted of the head, three thoracic segments, and ten abdominal segments. The head, thorax, and abdomen were devoid of combs, and the posterior margins of the thoracic and abdominal tergites lacked terminal spines (Figure 2).

**Figure 3:**
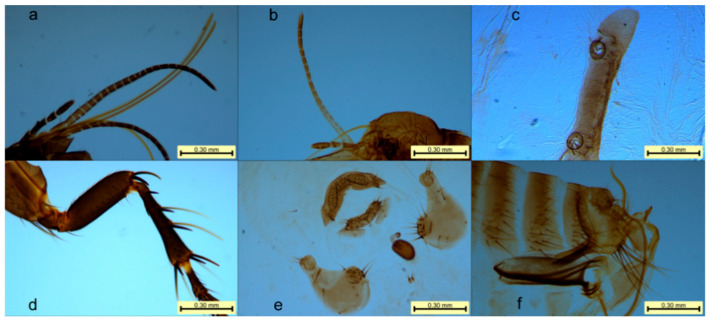
Partial local morphology of Vermipsyllidae: (**a**) labial palps of female Vermipsyllidae; (**b**) labial palps of male Vermipsyllidae; (**c**) spiracles of Vermipsyllidae; (**d**) hind tibia of Vermipsyllidae; (**e**) modified genitalia and spermatheca of female Vermipsyllidae; and (**f**) modified genitalia and apex of the aedeagus of male Vermipsyllidae.

Head: The head lacked a frontal suture. The antennae were located behind the eyes and were distant from the vertex of the head. They were composed of the scape, pedicel, and flagellum. The pedicel had long setae at the end, and the flagellum was annular, consisting of nine segments. The eyes were large with an inner fossa, and there were three ocular setae. The piercing–sucking mouthparts were composed of a labium, a hypopharynx, and one pair each of labial setae, maxillary setae, maxillary lobes, and maxillary inner lobes. The hypopharynx was a short basal plate located at the bottom of the mouthparts, bearing the labial setae. The females had 20–22 segments (Figure 3a), whereas the males had 17–19 segments (Figure 3b), which significantly exceeded the length of the first tibial segment. Each segment had one small spine. The maxillary setae consisted of four segments with many small setae. The hypopharynx was elongated and needle-like, and the maxillary inner lobes were located on both sides of the hypopharynx. The mid-lower segment of the inner lobes had backward-facing teeth, and the maxillary inner lobes were triangular and small, consisting of bony fragments.

Thorax: The thoracic segments were differentiated into the prothorax, mesothorax, and metathorax, with each segment having a dorsal plate bearing setae. Each thoracic segment had one pair of legs: the forelegs, middle legs, and hind legs. Each leg consisted of five segments: the coxa, trochanter, femur, tibia, and tarsus. The hind tibia had four notches along the posterior margin (Figure 3d), with more than 20 small setae on the lateral surface. The first tarsal segment lacked notches.

Abdomen: The eighth and ninth abdominal segments of males and the seventh to ninth abdominal segments of females were modified into reproductive segments. The ventral plates of the abdominal segments were separated into two small parts along the midline, connected only by a thin membrane. The abdomen of the female flea swelled during the egg-laying period, and the intersegmental membranes extended, whereas the dorsal and ventral plates retained their original size and shape. The spiracle on the mid-dorsal plate was relatively round, with a diameter larger than that of the eye. On the eighth dorsal plate, three (±1) long setae were located below the spiracle, and there was a spiracle fossa with small spines inside (Figure 3c). The ninth abdominal segment of males extended forward as a stalk-like structure and backward as a clasping organ (also called the immovable spur). The clasping organ was connected to the stalk-like structure by a movable spur. The immovable spur on the clasping organ had three (±1) long setae on the lower posterior edge, and each side of the ventral plate had 13 (±1) cup-shaped depressions. The male stalk was slender, approximately five times longer than its width, with a large end and a long, curved internal tube (Figure 3f). The female fertilization sac was shaped like a mango, with the posterior end bent at a right angle. Both males and females lacked preanal setae, and females lacked an anal cone (Figure 3e).

### 3.3. Molecular Biological Result

**Figure 4:**
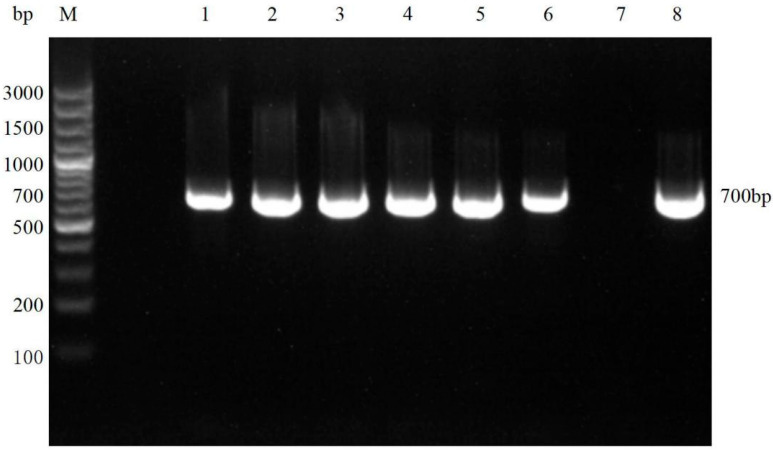
PCR amplification results for *D. ioffi* DNA: (M) DNA marker; (1–6) DNA samples of *D. ioffi*; (7) negative control; (8) positive control. Original image presented as Figure S1.

**Figure 5:**
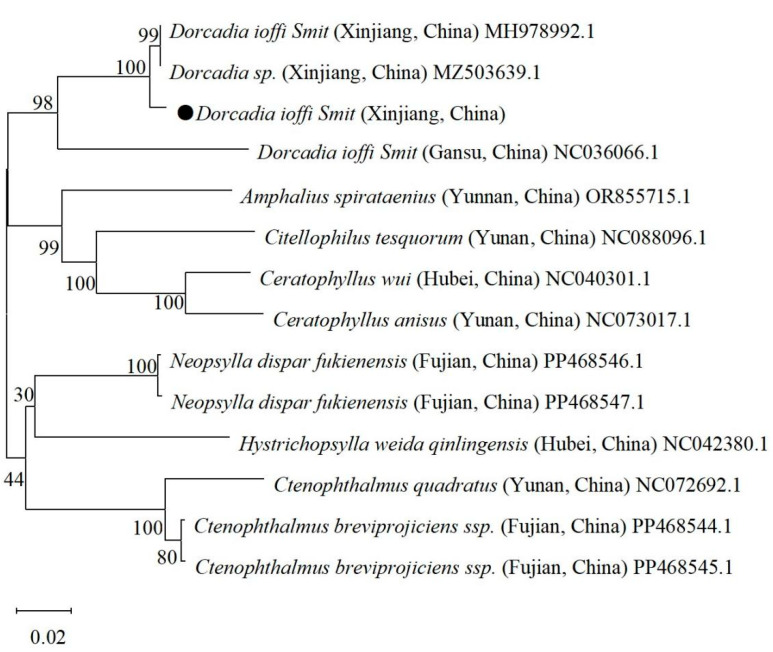
Phylogenetic tree analysis based on the SNP DNA sequences of *D. ioffi*; ● is SNP DNA sequences of *D. ioffi* used in this study.

In this study, the Vermipsyllidae DNA was successfully amplified to produce a fragment of approximately 700 bp, which was consistent with the expected size (Figure 4). Sequence alignment analysis revealed that the sequences of the six samples were identical. The sequence similarity compared with the *D. ioffi* sequences from sheep in Xinjiang, China (GenBank accession numbers MH978992.1 and MZ503639.1), was 99.13%. The sequence similarity compared with the *D. ioffi* sequences from sheep in Tianshui, Gansu Province, China, was 88.5%. Phylogenetic tree analysis revealed that the *D. ioffi* samples in this study clustered within the same evolutionary branch as those from Xinjiang and Gansu (Figure 5).

### 3.4. Statistical Analysis of Efficacy of Different Drugs

#### 3.4.1. *D. ioffi* Infestation Counts

**Figure 6:**
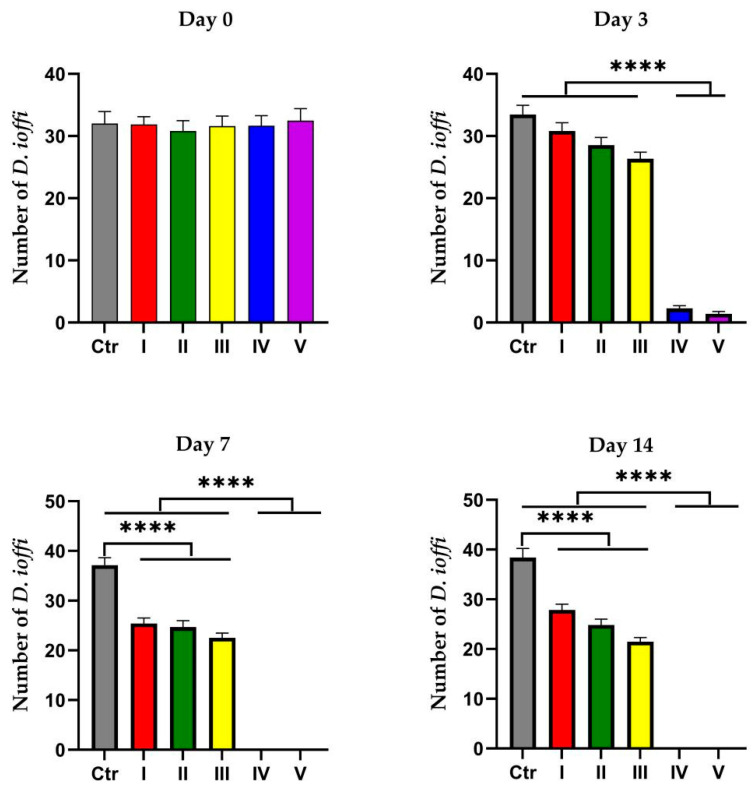
Statistical counts of *D. ioffi* infestation numbers in each medication group on Days 0, 3, 7, and 17 post-administration. Data are presented as mean ± standard deviation (mean ± SD). **** denotes *p* < 0.0001.Color coding: gray for Group Ctr, red for Group I, green for Group II, yellow for Group III, blue for Group IV, and purple for Group V.

Prior to administration (Day 0), no significant differences were observed in *D. ioffi* infestation counts among experimental groups (*p* > 0.05). On Day 3 post-administration, infestation counts in Groups IV and V were extremely significantly lower than those in the Ctr, I, II, and III groups (*p* < 0.0001). By Day 7, *D. ioffi* counts in Groups I, II, and III were extremely significantly lower than those in the Ctr group (*p* < 0.0001), whereas counts in Groups IV and V were reduced to zero—both groups showed extremely significant differences from the Ctr, I, II, and III groups (*p* < 0.0001). On Day 14, infestation counts in Groups I, II, and III remained extremely significantly lower than the Ctr group (*p* < 0.0001), while Groups IV and V sustained zero infestations. *D. ioffi* counts in Groups IV and V were extremely significantly lower than those in all other groups (Ctr, I, II, III; *p* < 0.0001) (Figure 6).

#### 3.4.2. Statistical Analysis of *D. ioffi* Population Reduction Rate

On Day 3, reduction rates in Groups III and IV were significantly higher than that in the Ctr group (*p* < 0.05), while Group V showed an extremely significant increase compared to the Ctr, I, II, III, and IV groups (*p* < 0.0001). By Day 7, reduction rates in Groups I, II, and III were extremely significantly higher than that in the Ctr group (*p* < 0.0001), whereas those in Groups IV and V were extremely significantly higher than in the Ctr, I, II, and III groups (*p* < 0.0001). Day 14 showed identical trends to those on Day 7 (Table 2).

#### 3.4.3. Therapeutic Cure Rate for *D. ioffi* Infection in Sheep

The results of cure rate statistics showed that on Day 3 after administration, the cure rates of Dorcadia ioffi in Groups I, II, III, IV, and V were 0, 0, 0, 0, and 26.67%, respectively. On Days 7 and 14, the cure rates of each group were 0, 0, 0, 100%, and 100%, respectively, for both time points. Throughout the experiment, with the increase in the number of days after administration, the cure rates of Groups I, II, and III remained 0 even on Day 14, while those of Groups IV and V reached 100% on Day 7, demonstrating the fastest onset of efficacy.

## 4. Discussion

The Vermipsyllidae family consists of three genera: *Chaetopsylla Kobaut*, *Vermipsylla Schimkewitsch*, and *Dorcadia Ioff.* Currently, in China, the identified species include 2 subgenera and 16 species of *Chaetopsylla Kobaut*, 10 species of *Vermipsylla Schimkewitsch*, and 5 species of *Dorcadia Ioff* [9,16]. In Xinjiang, the identified species include *Chaetopsylla Kobaut* species such as *Chaetopsylla (Arctopsylla) lasia*, *Chaetopsylla (Chaetopsylla) homoea*, *Chaetopsylla (Chaetopsylla) globiceps,* and *Chaetopsylla (Chaetopsylla) trichosa*; *Vermipsylla Schimkewitsch* species such as *Vermipsylla ibexa, Vermipsylla yeae*, and *Vermipsylla alakurt*; and *Dorcadia Ioff* species such as *D. ioffi* and *Dorcadia dorcadia* [17]. This study identified the prevalent Vermipsyllid species in the Altay region as *D. ioffi* through morphological observation and molecular biological analysis and compared the anthelmintic effects of different drugs via pharmacodynamic experiments. Cypermethrin and dichlorvos were screened out for their high anthelmintic activity.

In the present study, morphological examination of the samples revealed the following characteristics of the Vermipsyllidae family: the flea head lacks a frontal suture, the antennae cavity does not reach the top of the head, no central bar is present, and there are no combs on the thorax. Neither sex has preanal setae; the female lacks a postanal spine and has only one fertilization sac. The morphological characteristics of the genus *Dorcadia* include large eyes with an inner sinus, three rows of eye setae, long maxillary palps, no cuticular ridges on the hind tarsus, and a broad penis with a long, curved internal tube, with females retaining their original abdominal size and shape even when engorged with many eggs. The characteristics specific to *D. ioffi* include female maxillary palps with 20–21 segments, male maxillary palps with 16–19 segments, four cuticular ridges on the hind tibia with two rows of setae on the exterior, and the dorsal spiracles of the intermediate abdominal segments being larger than the eyes. These findings align with the descriptions by Zhao C G et al. [18] and Liu Q [19].

Molecular biological identification revealed that the primary flea species in the Altai region is *D. ioffi*, exhibiting high similarity (99.13%) with the reference sequences of *D. ioffi* from Xinjiang (GenBank accession numbers: MH978992.1, MZ503639.1), further confirming the population consistency of this species. This suggests that the *D. ioffi* population in Xinjiang may have a relatively fixed genotype structure. However, the genetic sequence similarity among the *D. ioffi* samples from Tianshui, Gansu, and Xinjiang was only 88.5%, indicating that geographical isolation may have caused genetic differentiation among the *D. ioffi* populations from different regions. This genetic variation could be related to regional ecological environments and host selection pressures [20]. Phylogenetic tree analysis revealed that the *D. ioffi* samples from Xinjiang and Gansu clustered together, suggesting that the populations in these regions may share a common evolutionary background. The clustered distribution of *D. ioffi* in Xinjiang could be related to the ecological environment of pastures and grazing methods, which also provides a theoretical basis for regional pest control strategies in the future.

The ideal temperature and humidity of highland pastures provide optimal conditions for the development of Vermipsylla species. Previous reports have indicated that the altitude range for Vermipsylla in the Qinghai and Xinjiang regions is mostly between 1800 and 4500 m [9,21]. However, in this study, *D. ioffi* was found in winter pastures at an altitude of 1500 m. These findings suggest that the adaptability of this species may extend beyond the previously reported altitude distribution range. This could be linked to climate change, as Li, D. [22] reported that global warming has caused shifts in the latitudinal and altitudinal ranges of insect species. This mechanism may also be reflected in the distribution of *D. ioffi* in the present study Zhang L et al. [23] noted that rising temperatures might cause some insect species to expand their distribution ranges toward lower latitudes and altitudes. The increased temperatures may have facilitated the survival and reproduction of *D. ioffi* at lower altitudes, causing a shift in its distribution range. Additionally, anthropogenic activities and the use of highland pastures may have altered the habitat of *D. ioffi*, creating a new ecological environment conducive to its survival, thus expanding its distribution range to lower altitudes.

Avermectin injection, ivermectin injection, and moxidectin spraying are endectocide antiparasitic drug treatments. The mechanism of action of these drugs involves binding to γ-aminobutyric acid (GABA) receptors in the parasite’s nervous system, which enhances the opening of chloride ion channels and leads to nervous system dysfunction, muscle paralysis, and, ultimately, the death of the parasite [24]. Bifenthrin works by interfering with the sodium ion channels in the parasite’s nervous system, causing persistent depolarization of nerve cells, excessive excitation, and paralysis, ultimately leading to parasite death [25]. Carbaryl inhibits cholinesterase activity in the parasite, preventing acetylcholine breakdown, causing the accumulation of neurotransmitters, and resulting in excessive excitation of the parasite’s nervous system, leading to paralysis and death [26].

The poor effectiveness of macrocyclic lactones in this study may be attributed to the emergence of resistance in local *D. ioffi* populations due to prolonged use of these drugs, thus reducing their efficacy. Additionally, this may be related to the biological characteristics of *D. ioffi*, such as its life cycle and sensitivity to drugs [27]. While carbaryl and bifenthrin have proven effective, their toxic side effects and safety should also be considered. Care should be taken when these drugs are used on young, pregnant, or weak sheep. When administering drugs, it is important to select the appropriate molecules and administration method, livestock species, and conditions, strictly following the instructions for the drug to ensure effective parasite control while ensuring livestock safety. Unnecessarily increasing drug dosages should be avoided to prevent the development of resistance.

## 5. Conclusions

This study indicates that the dominant flea species prevalent in the Altai region of Xinjiang is *D. ioffi*, a member of the Vermipsyllidae family. This species is closely related to the *D. ioffi* populations reported in Xinjiang and Gansu Provinces. The most effective drugs are carbaryl and bifenthrin, with the recommended method of administration being pour-on. The suggested doses are 0.1 mL/(kg·BW) carbaryl and 0.2 mL/(kg·BW) bifenthrin.

## Figures and Tables

**Table 1:** Experimental grouping and administration.

Group	Treatment	Dose
Ctr (untreated control group)	Physiological saline	0.2 mL/(kg·BW)
I (avermectin injection group)	Subcutaneous injection	0.2 mg/(kg·BW)
II (ivermectin injection group)	Subcutaneous injection	0.2 mg/(kg·BW)
III (moxidectin pour-on group)	Pour-on along the dorsal midline	0.05 mL/(kg·BW)
IV (deltamethrin solution pour-on group)	Pour-on along the neck, abdomen, and chest	0.1 mL/(kg·BW)
V (trichlorfon pour-on group)	Pour-on along the neck, abdomen, and chest	0.2 mL/(kg·BW)

**Table 2:** Statistical data on the *Dorcadia ioffi Smit* population reduction rate across different group.

Group	Time (d)		
	3	7	14
Ctr	−9.3 ± 7.86 ^cdF^	−22.88 ± 9.21 ^BCDEF^	−27.12 ± 10.08 ^BCDEF^
I	1.71 ± 5.13 ^F^	18.86 ± 4.60 ^AEF^	10.86 ± 4.81 ^AEF^
II	3.99 ± 5.70 ^F^	15.94 ± 7.43 ^AEF^	15.76 ± 6.36 ^AEF^
III	14.24 ± 4.79 ^aF^	26.50 ± 4.34 ^AEF^	30.13 ± 3.68 ^AEF^
IV	13.59 ± 5.87 ^aF^	100.00 ± 0 ^ABCD^	100.00 ± 0 ^ABCD^
V	95.59 ± 1.18 ^ABCDE^	100.00 ± 0 ^ABCD^	100.00 ± 0 ^ABCD^
*p*	<0.0001	<0.0001	<0.0001

Note: For data in the same column, one-way ANOVA was performed, followed by multiple comparisons for each group. Superscripts “a” and “A” denote significant (*p* < 0.05) and extremely significant (*p* < 0.01) differences compared with the Ctr group, respectively; “B” indicate significant (*p* < 0.05) and extremely significant (*p* < 0.01) differences compared with Group I; “c” and “C” represent significant (*p* < 0.05) and extremely significant (*p* < 0.01) differences compared with Group II; “d” and “D” signify significant (*p* < 0.05) and extremely significant (*p* < 0.01) differences compared with Group III; “E” denote significant (*p* < 0.05) and extremely significant (*p* < 0.01) differences compared with Group IV; and “F” indicate significant (*p* < 0.05) and extremely significant (*p* < 0.01) differences compared with Group V.

## Data Availability

All data is contained within the paper and its Supplementary Materials.

## Supplementary Materials

The following supporting information can be downloaded at: https://www.mdpi.com/article/10.3390/vetsci12070641/s1, Figure S1: original gel images.
