# Supplementary material for: Identification of the Pathogen Dorcadia ioffi Smit and Evaluation of the Effect of Different Drugs
Source: Vet Sci. 2025 Jul 4;12(7):641. doi: 10.3390/vetsci12070641 (PMC12297897; doi:10.3390/vetsci12070641)
Supplement: Supplementary file 1 [file vetsci-12-00641-s001.zip › vetsci-3691086-supplementary.pdf]

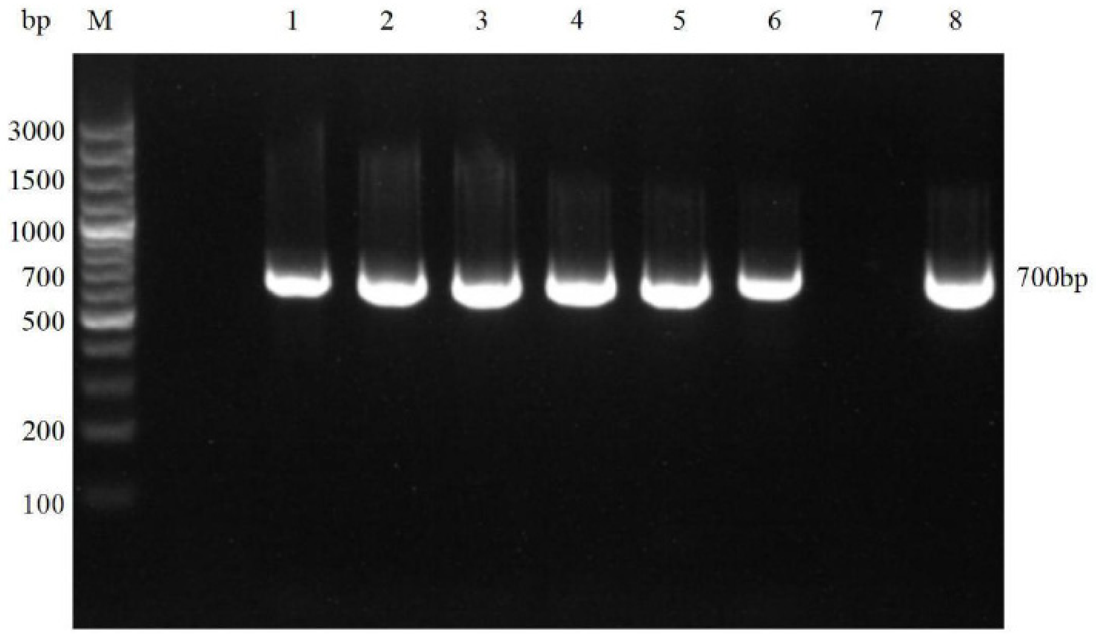

Figure S1: Original Image of PCR amplification results for Vermipsyllidae DNA: (M) DNA marker; (1~6) DNA samples of Vermipsyllidae; (7) negative control; (8) positive control.
